# Pneumococcal Peritonitis

**Published:** 1902-12-27

**Authors:** 


### PNEUMOCOCCAL PERITONITIS.

The rôle of the pneumococcus in pathology is both obscure and important. It is always present in certain lesions, apparently alone in many cases, and when injected into animals it reproduces the symptoms of the disease. Yet, as is well known, it is present in the mouths of perhaps one healthy person out of every four. Does it under certain circumstances acquire virulence, or is it always pathogenic, but only producing injurious effects when the heart suffers from some shock or depressing circumstances? Supporters have arisen for either theory, and the final answer has not been given. The same difficulty is met with in considering the rôle of other micro-organisms, though the question with them takes slightly different forms. Some are found outside the body, as well as in a virulent parasitic form, others, such as the streptococcus, appear to be the cause of so many affections that it is hard to believe that we have to do with only one species. However the question of the double life of the pneumococcus may be settled, no one doubts its activity in lobar pneumonia, but of late evidence has accumulated against it in many other directions. First it was found in pneumococcal meningitis, then some cases of pericarditis and empyema were found to give pure cultures of this diplococcus. It was said that this was natural when pneumonia preceded these attacks, because the organism was carried by lymphatics to the neighbouring serous sacs. These pleural effusions, too, were different in type from strepto, staphylococcal or tubercular ones, and tended more directly to recovery. Some writers went so far as to say that an empyema of this class would disappear after simple aspiration, and this seems to be occasionally the case.

Joint affections were next noticed, presenting pure cultures of the diplococcus and going on to rapid destruction of the articulation, and here the question of the mode of entrance becomes less simple. If introduced from the blood stream, how is it that so few cases of pneumonia show the diplococci in the blood? It may be argued that ordinarily they are present only as stragglers, or at a particular stage of the disease, and are quickly destroyed. Again, the mucous membranes are occasionally affected with lesions in which this diplococcus is present either alone or, at least, in an overwhelming majority. Such are some cases of membranous pharyngitis and gastritis, tonsillar abscesses, and ulceration of the stomach and bowels. In many of these diseases there is no history of a previous pneumonia, but under some unknown conditions the diplococcus becomes able to multiply and destroy the tissues in widely distant regions of the body.

An interesting type of these invasions is seen in certain cases of peritonitis, though it is not long ago that Treves hesitated to believe that any were due to the diplococcus alone. However, a considerable number have been observed of late, some showing a pure culture just as the cases of pneumonia and arthritis do. One writer alone collected 40 instances of which 34 were in young girls. Indeed, comparatively few are recorded in adults, and even cases in boys are not numerous. Thus Bryant saw two girls and one man attacked, and he quotes Quehant as describing twenty-seven cases in girls and six in boys. As to this fact Brun and others believe that the infection here enters through the uterine tract, for the organism has been found in the uterus, and when the peritonitis is a localised one, the site is nearly always in the pelvic region. Still there must be other channels, in male patients for instance, and these may be the lymphatics from the lungs and pleuræ, or the blood stream if a septicæmia exists, or the intestinal tract. Probably the course varies and is not always the same. Some of these instances of pneumococcal peritonitis have been primary, others have followed otitis, lung affections, appendicitis, ovarian disease, and pelvic neoplasms.[1]

In pneumococcal peritonitis the patient may be taken ill with abdominal pain, diarrhœa, and vomiting. Appendicitis may be first thought of, and distension and tenderness in some part of the abdomen may be marked for a time. Possibly some lung symptoms may follow and lead us to suppose that here is a pneumonia ushered in by abdominal symptoms of a transient kind. However, in a few days, the abdomen generally becomes more distended and contains fluid which may be sero-fibrinous or purulent. Indeed the whole cavity may be full of a thick creamy pus, though Bryant thinks that it is usually thin and that it separates on standing into two layers, a lower one containing the more solid matters, and the upper one, which is three-quarters of the whole, clear and limpid. The collection may burst its way through the umbilicus or elsewhere, if the patient lives long enough and is not relieved by surgical aid. It may be encysted in one part of the peritoneum or may be diffused over the whole. If opened and drained the mortality does not seem to be very high. Of Brun's 14 cases, 11 recovered, ten of them after operation and one after the formation of a sinus. Cave, of Bath, also records a successful case, but the three discussed by Bryant died. Whether recovery would take place after aspiration alone is uncertain, but a general diffuse peritonitis is too serious and dangerous a malady for half measures. When it is due to other forms of infection very few cases recover however thoroughly they are treated, and it would hardly be right to lose time in trying aspiration because a few cases of pneumococcal empyema have cleared up under that treatment. Yet it is possible that this extraordinary affection may not after all be very rare, and that many obscure cases of peritonitis which show only a trifling effusion and end in spontaneous recovery may eventually turn out to be of this type; for it is probable that the recorded cases have been chiefly those where the effusion or the constitutional symptoms were marked and that mild ones have been unrecognised. Looking at the number and variety of pneumococcal affections, the wonder is that the peritoneum escapes so often and that peritonitis is not a common complication of the diseases which are due to the organism in question.

[1] Brit. Med. Jour., Sept. 21, 1901.

